# Successful ageing in the oldest old: objectively and subjectively measured evidence from a population-based survey in Germany

**DOI:** 10.1007/s10433-021-00609-7

**Published:** 2021-03-29

**Authors:** Marina Plugge

**Affiliations:** Faculty of Management, Economics and Social Sciences, Institute of Sociology and Social Psychology (ISS), Graduate School GROW – Gerontological Research on Well-Being, University of Cologne, Albertus-Magnus-Platz, 50923 Cologne, Germany

**Keywords:** Successful ageing, Subjective well-being, Oldest old

## Abstract

Despite rapid increase of people aged 80 and over, concepts of successful ageing (SA) are primarily examined for people below that age. Therefore, successful ageing was examined in a population-based representative sample of *N* = 1863 people aged 80 to 102 (NRW80+) with 11% living in institutionalized settings. In this survey on quality of life and well-being, multiple linear and logistic regression models were used to calculate the distribution of successful agers. According to Rowe and Kahn’s objective definition, 9% of the sample aged successfully, but one-third (33%) still met four to five SA criteria. This is in line with the theoretical a priori criterion of 10% in a normal distribution of a sample, while 80% age normally and 10% pathologically. However, averages of life satisfaction, affective well-being, positive ageing experience and valuation of life were high. The majority of the oldest old (65%) are successful agers in their own subjective perception, which is not in line with objective measurements. Moreover, 11% of objectively measured successful agers do not meet subjective criteria. These empirical findings reveal a remarkable discrepancy between objective and subjective criteria of SA. Future research on concepts that define successful ageing for the oldest old should consider more holistic markers of success, e.g., outcomes of productive social engagement.

**Supplementary Information:**

The online version contains supplementary material available at 10.1007/s10433-021-00609-7.

## Introduction

The concept of successful ageing (SA) as proposed by Rowe and Kahn (1997) aimed at shifting the perspective from a deficit to a resource-oriented focus on ageing to overcome the dichotomy of pathological and normal ageing. This well-known, likewise probably most criticized concept (Cosco et al. 2014; Katz and Calasanti 2015; Manierre 2018; Martinson and Berridge 2015) is based on objective criteria. It describes SA as absence of chronic diseases or illness-related impairments while maintaining cognitive and physical functions and an active lifestyle (Rowe and Kahn 1997, 2015). Considering these criteria for different age groups and the ageing process as a whole, the following questions need to be asked: How long can this “successfulness” be preserved and which prerequisites are necessary for it? And should success be measured based on objective criteria only?

These questions are of special interest for the fourth age, which is characterized by health restrictions and the need for care (Kruse 2017; Smith and Ryan 2016). However, this group of oldest old scores well in subjective criteria, e.g., constructs of subjective well-being (Jopp et al. 2008). This unexpected discrepancy between objective and subjective SA indicators might be explained by approaches of adaptation process of ageing, e.g. *selection–optimization–compensation theory* according to Baltes and Carstensen (1996) or *two-process model* according to Brandtstädter and Renner (1990). Common features of these theories are strategies of older people and their adaptability to deal with negative age-related influences. This means that older people develop skills in their life-course for the successful adaptation to changes and demands (Jopp et al. 2016; Nikitin and Freund 2019).

Although directly arisen from the criticism that was brought forward in a long debate about deficits of the oldest old, the concept of SA has hardly been examined in this age group. Differentiated analyses can only be made for people aged 65 to 80, while research on SA has too small sample sizes of people aged 80 or over (Baker et al. 2009; Bosnes et al. 2017; Dahany et al. 2014; Hank 2011; Li et al. 2014; Whitley et al. 2018). However, this age group demands more attention due to its rapid growth. In Europe, their proportion of the population will double in 2070 (Eurostat 2018). In Germany, about one in eight people will be 80 or older by 2060 (Statistisches Bundesamt 2015). The age limit which defines individuals to be very old is not clearly defined (Motel-Klingebiel et al. 2013). Despite the lack of a uniform, cross-disciplinary definition for determining very old age, the categorisation for the oldest old with a limit of 80 years in contrast to 60 years for the young old serves as an orientation for research and practice (Baltes and Smith 2003; Kruse 2017).

Based on these considerations, this paper addresses these two specific research questions:

(I) Is successful ageing as defined by Rowe and Kahn’s objective criteria still possible from the age of 80 years up? The key contribution is to analyse the SA model by Rowe and Kahn (1997) with a dataset of the oldest old.
(II) Are prevalence rates for successful ageing in old age higher if the criteria are defined subjectively rather than objectively? This will contribute to gaining a more holistic perspective on SA and will build on the critiques of Rowe and Kahn’s concept by Cosco et al. (2014) and Martinson and Berridge (2015).

The empirical analyses to answer these questions use the representative data set of very old people in Germany (NRW80+ , *N* = 1.863). This study enables to determine the distribution of SA for a population aged 80 and over in detail and to compare analyses of subjective and objective criteria for the first time. The sample is characterized by an overrepresentation of nursing home residents (Wagner et al. 2018b). This group is often excluded from population surveys due to institutional or personal access barriers (Schanze 2017). Consequently, assumptions might be distorted since the residents’ specific life situation and perspectives can lead to a lack of participation (Kelfveet et al. 2013; Wagner et al. 2018a).

### Concept and theoretical anchoring of SA in the oldest old

The MacArthur model of SA developed by Rowe and Kahn (1997) was a starting point to overcome the dichotomy of pathological and normal ageing. While this deficit-oriented classification had dominated the field before, they wanted to take a resource-rich perspective instead. Therefore, Rowe and Kahn proposed to apply a normal distribution and differentiate between three types of ageing:10% *pathological*, 80% *normal* and 10% *successful* agers.

According to these objective criteria, individuals are successful agers if they have (1) no chronic diseases and disabilities, (2) high cognitive and (3) physical functions and if they are (4) interpersonally and (5) productively integrated (Rowe and Kahn 1997, 2015). This approach was severely criticized (Cosco et al. 2014; Katz and Calasanti 2015; Manierre 2018; Martinson and Berridge 2015). In particular, challenges such as the onset of the need for care due to health restrictions can influence the social inclusion of the oldest old. Incorporating the criticism, Rowe and Kahn (2015) adapted their SA model while focusing on macrosocial structures, i.e., considering the ageing process in a societal context with remarkable influences on social contacts (Rowe and Kahn 2015). Several concepts have been proposed to better understand the consequences of restrictions in health and functional capacity on leading independent and satisfying lives, some of which refer explicitly to very old age.

The CHAPO (*The Challenges and Potentials*) model, in extension of the model of Veenhoven (2000), explicitly suggests including a conceptual domain of successful life conduct in order to address person-environment constellations in the oldest (Wagner et al. 2018b). Many concepts of successful life conduct are characterized by both the internal value system of the individual as well as the normative values given by the environment. With regard to life achievements, a successful lifestyle can be described, for example, through the degree of social integration (Veenhoven 2000; Wagner et al. 2018b). The model displays that quality of life must be viewed both holistically and subjectively. The latter is represented by the aspect “appreciation of own life” (Wagner et al. 2018b), in which life satisfaction and affective and psychological well-being play a central role.

Consequently, successful life conduct as introduced by Wagner et al. (2018b) may be experienced as fulfilling for the individual but also appreciated by others. This provides the possibility of integrating both the individual-focused traditional concept of SA proposed by Rowe and Kahn (1997) as well as the macrosocial perspective suggested by Tesch-Römer and Wahl (2017). They describe a model which includes those who grow old with disabilities and care needs (Tesch-Römer and Wahl 2017). The model presents individual, environmental and care-related strategies for autonomy and quality of life and emphasize inter-individual differences and social inequality in old age. The analyses carried out here consider comparisons between individuals in institutionalized versus private settings plus demographics that might reinforce social inequality.

### Distribution of SA in the third age

Research on SA has been conducted in Europe (Bosnes et al. 2017; Dahany et al. 2014; Hank 2011; Whitley et al. 2018), in the USA (McLaughlin et al. 2010) and in Asia (Nakagawa et al. 2020). However, it has not yet been possible to examine the concept empirically and apply it to very old individuals due to incompleteness, complexity of the Rowe and Kahn model and lack of available data (Dahany et al. 2014; Whitley et al. 2018). A few studies consider the oldest, but these cannot differentiate between people aged under or over 80 years (Baker et al. 2009; Bosnes et al. 2017; Hank 2011; McLaughlin et al. 2010). Consequently, previous analyses of SA only apply for people in the third age, which ranges from 65 to 80 years (Baltes 1999).

In Germany, the proportion of successfully ageing people in the third age is 12%, while the overall average of 14 European countries and Israel is 8.5% (Hank 2011). Cross-sectional studies of Canada show a proportion of 11% successful agers from 60 years up (Baker et al. 2009). This is very similar to the results of longitudinal studies of the United States by McLaughlin et al. (2010) with a prevalence rate of 12%. By contrast, a French sample incorporating age ranges only from 65 up to 75 years shows higher proportions with 30% successful agers (Dahany et al. 2014). This is in line with the result of a systematic review which found an average proportion of 26% in the United States, Canada, United Kingdom, Australia, and counting (Cosco et al. 2014). Apart from that, gender differences were examined in Korea, resulting in prevalences of 14% and 9% for men and women, respectively (Kim et al. 2019).

Although these findings provide an orientation for the worldwide prevalence rate of SA in the third age, they are not sufficiently comparable due to different sample sizes, age limits and methods. Therefore, it is even more difficult to draw conclusions for the fourth age. Due to their declining health status, it can be assumed that the proportion of SA is lower in people aged 80 and over compared to younger samples. Hence, for the analysis of a German sample of oldest old, a SA proportion lower than 12% is to be expected.

## Methods

### Data

The analyses were conducted using the first wave of data collection from the representative German “Survey on quality of life and subjective well-being of the very old in North Rhine-Westphalia (NRW80+)”, the most populous federal state of Germany (Wagner et al. 2018b). The study goal was to establish a database for the oldest old to explain differences in quality of life outcomes. This cross section provides information about demographics, the material, social and health status, life style, values and attitudes of individuals aged 80 to 102 years. The sample consisted of 1863 interviews drawn from registration offices of 94 municipalities in the state North Rhine-Westphalia. Approximately 10% constituted proxy interviews with relatives or caregivers for individuals who were unable to participate and 11% of the interviews were conducted with institutionalized respondents (see Table 1). To contrast the relevant subpopulations, oversampling was performed for persons from institutionalized settings, for men, and for persons in the oldest age segment. For this reason, the proportion of male is 50%, although the proportion of women outnumbers men in this age segment.

**Table 1:**
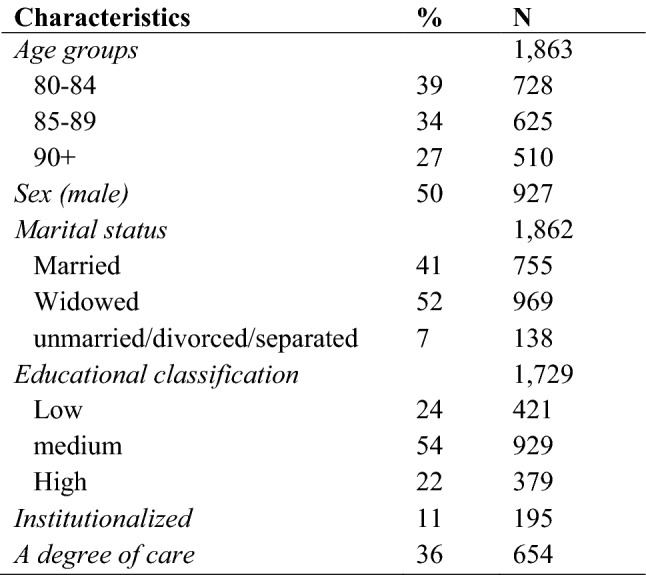
Sample characteristics

Unweighted data with N = 1863

The data were generated through computer-aided personal interviews with an average duration of 1.5 h. This instrument has been developed at the Center for Ethics, Rights, Economics and Social Sciences of Health (ceres) at the University of Cologne. The study was approved by the Research Ethics Committee at the University of Cologne (17–169). Data collection took place between August 2017 and February 2018 with a response rate of 23% (Wagner et al. 2018a). Both the objective and subjective indicators of SA (see “Variables” section) were measured by this unique data of the oldest old.

### Variables

#### Objective criteria of SA

Traditionally, SA has been assessed based on the definition of Rowe and Kahn (1997), but different researchers have measured it in a variety of ways. There is no consistency in the definition of SA (Cosco et al. 2014). This study provides a post hoc definition by replicating the operationalization of three major recent European ageing studies: the Nord-Trøndelag Health Study (Bosnes et al. 2017), SHARE (Hank 2011), and the West of Scotland Twenty -07 cohort study (Whitley et al. 2018). The results of a construct validity study about the operationalization using confirmatory factor analysis showed best model fit if the dependent variable SA included all dimensions described by Rowe and Kahn’s concept. In addition, it is recommended to allow differences between the single dimensions (Kleineidam et al. 2019).

Hence, SA is a binary indicator that equals 1 if all five dimensions are fulfilled and 0 if not. The dependent variables consist of this overall indicator and its individual binary coded components. To make the operationalization as transparent as possible and in order to show differences depending on the researcher’s subjective election of variables, four overall indicators by different coding schemes were applied (see Table 2).

**Table 2:**
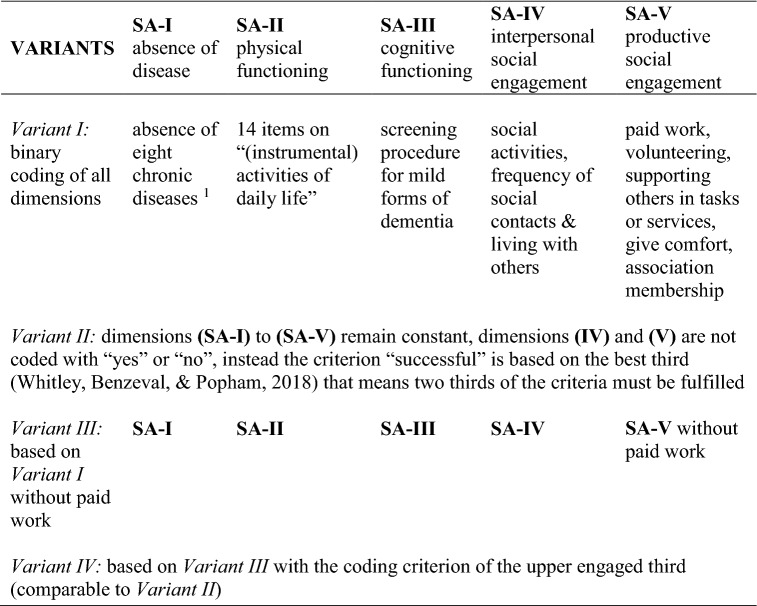
Overview of different variants on indicators to measure objective criteria of SA

For further details about instruments, operationalization and distribution see Table S1 in the Online Appendix

^1^Coronary heart disease, stroke, chronic obstructive pulmonary disease (COPD), cancer (skinless), diabetes, Parkinson's disease and depression

#### Subjective criteria as indicators for SA

This operationalization aims to describe how success can be measured by subjective criteria from the perspective of the 80+ population. In fact, in this population, subjective criteria seem far more valuable to determining success because chronic diseases are exceedingly common. However, this is only an issue if it interferes with one’s ability to engage in a lifestyle that is satisfying and meaningful. The subjective assessments can help highlight the ways to identify which older people in this phase of the life course are thriving. The construct “appreciation of own life” (Wagner et al. 2018b) can be operationalized by life satisfaction, affective and psychological well-being. Processes of staying attached or connected to life may become increasingly important in light of diminishing resources in the oldest old (Wahl et al. 2012). This attachment is operationalized by ageing experience. Various measurements of subjective perceptions were conducted (see Table 3). In order to gain an overall indicator of the subjective perception and to draw comparisons with objective criteria, the very satisfied and those with very positive ageing experience were summarized in one indicator.

**Table 3:**
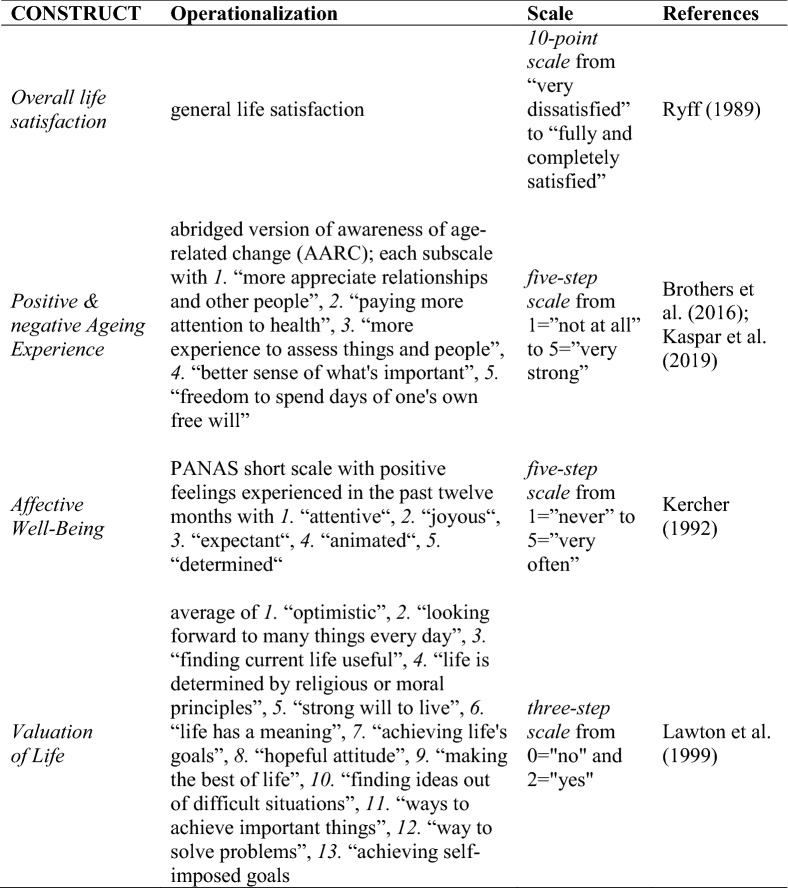
Overview of subjective criteria as indicators for SA

#### Independent variables

The statistical analyses are controlled for socio-demographic variables, namely age, sex, marital status, and education. Age was categorized in three groups (80–84 years, 85–90 years and 90+). The reason to split the sample into these groups is due to approximately the same size of proportion (see Table 1). Furthermore, age is a very interesting variable to analyse the success of ageing by itself. To get more detail about the success of each ageing group it is necessary to examine if there is a difference between these groups. The marital status was divided in married, widowed, divorced, and unmarried. Education was separated into the categories low, medium, and high. Low includes respondents without any completed vocational training and with a secondary school leaving certificate or lower. The category medium education comprises individuals with completed vocational training or a university entrance qualification. High level includes respondents who have completed their studies.

### Statistical analysis

The analyses test the empirical evidence for the assumption that different ways of living affect how “good” life is as perceived by the very old themselves. First the distribution of successful agers and their five domains were calculated. Multivariate logistic regression analyses were used to assess the independent effects of each variable’s ability to explain SA through objective markers. The estimates are presented as odds ratios (OR) with 95% confidence intervals (CI). Second, rates for subjective definitions of SA were calculated (see “Subjective criteria as indicators for SA” section). Moreover, multivariate linear regression analyses were conducted with subjective perceptions as dependent variables.

To account for unit nonresponse, the population weight for North Rhine-Westphalia was applied in the descriptive analysis of the distribution of successful agers. For regression analyses, personal calibration weights were calculated to obtain undistorted estimates. The cluster structure at municipal level was additionally used for the purpose of undistorted estimates in the regression analyses. The original sample size of 1863 respondents is reduced to 1413 in logistic regressions and to 1658 in linear regressions as there are missing values for dementia diagnosis and subjective evaluation criteria of proxy interviews. Statistical significance was set to *p* < 0.05 by analyses with Stata (version 16). Reliability was investigated using Cronbach’s alpha.

## Results

### Bivariate analyses of objective and subjective criteria for SA

The distribution of SA is shown in Table 4. In the first variant with binary measurement, 9% age successfully, which means that all five criteria are met, while 2% do not meet any of the criteria. In between, 89% fulfil one to four criteria, while one-third meets four to five criteria. The following distribution rates can be calculated for the five criteria: Almost two-thirds of the participants show good cognitive functioning and nearly all have a high level of interpersonal social participation. Productive social engagement is present in two-thirds of cases. Finally, one-third has good physical functionality and no chronic diseases.

**Table 4:**
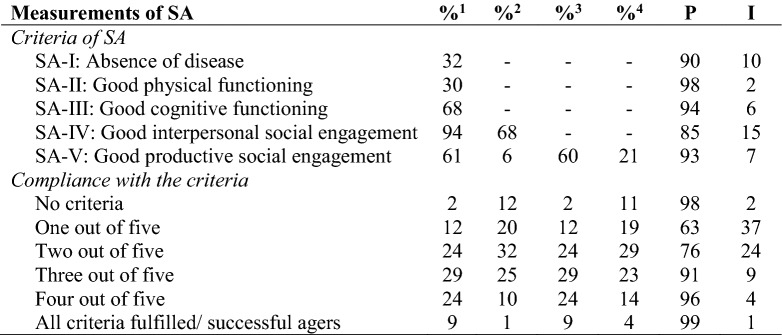
Distribution of objectively measured criteria of SA in the oldest old

P private housing type, I institutionalized housing type, N = 1.863; weighted data with population size N = 1,077,296

^1^Binary coding for all five dimensions

^2^Upper third measurement concerning dimension social engagement

^3^Without criterion “paid work” concerning dimension productive social engagement (binary measurement)

^4^Without criterion “paid work” concerning dimension productive social engagement (upper third measurement)

In the second variant where the upper third of social participation is considered as successful, there are clear deviations from variant one. According to this measurement, interpersonal social engagement is high for two-thirds in contrast to the previous proportion of 94%. A high level of productive social participation is shown for only 6% instead of 61%.

60% are rated as good agers following variant three which excludes paid work as a criterion for productive social participation. This is comparable to variant one (61%). Variant four which does not consider paid work and with the best third has a much lower value than variant three and a much higher value than variant two (21% vs. 60% vs. 6%). A huge difference between private and institutionalized settings is shown by comparisons of successful agers (99% vs. 1%). This finding is equivalent in all five criteria and evident in the compliance with these criteria.

The first set of analyses of subjective criteria as indicators for SA (see Table 5) showed high average levels of life satisfaction (79%). The distribution of positive ageing experience supports the hypothesis of high subjective perceptions, with more than half rating their ageing experience as positive and even more than a third as very positive. The mean values of positive and negative ageing experiences are high, too (3.2 on a five-point scale). Comparisons between private and institutionalized settings show small differences, but these are not as high as in objective measurements (see Table 4). The mean values of ageing experience, affective well-being and valuation of life are even smaller in institutionalized settings than in private residents (see Table 5).

**Table 5:**
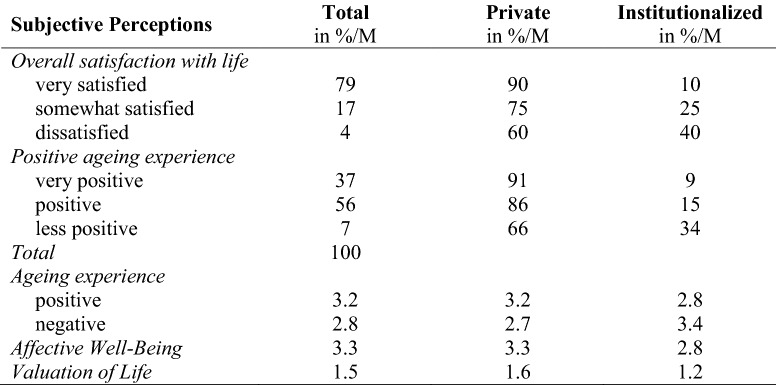
Distribution of subjective perceptions of SA in the oldest old

Weighted data with population size N = 1,077,296; scale of “positive and negative ageing experience” from 1 = “not at all” to 5 = “very strong”; scale of “affective well-being” from 1 = “never” to 5 = “very often”; scale of “valuation of life” from 0 = “no” to 2 = “yes”; M = Means

There are two further measurements of subjective well-being: The mean values of the PANAS short scale show that with a scale value of 3.3, most older individuals have “sometimes” to “often” experienced positive feelings in the past twelve months. The average value of valuation of life is with 1.5 on a scale from zero to two rather high.

When drawing comparisons between the measuring modes of success on ageing, there is evidence of a discrepancy: More than two-thirds are subjectively measured successful agers, but this proportion is not confirmed by objective measurements. By contrast 11% of objectively measured successful agers do not meet subjective criteria (see Table 6).

**Table 6:**
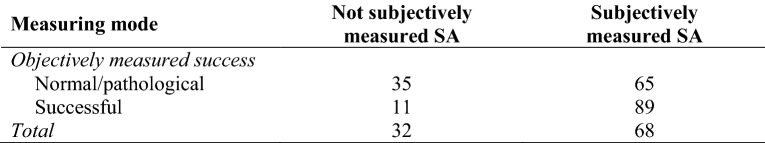
Objectively measured versus subjectively measured criteria of SA

Weighted data with population size N = 1,077,296

### Multivariate analyses of objective and subjective criteria for SA

The results of the multivariate logistic regression analyses are presented in Table 7. The first binary-coded regression analysis shows that younger age and a higher degree of education significantly correlate with SA. By contrast, gender and marital status show no significant influence. The regression analysis of variant two does not indicate any presentable results since the dependent variable with only 2% of successful agers shows too little variance. Variants three and four do not differ from variant one in regarding age, sex, and marital status. However, the coefficient of higher educational attainment in variant four is twice as high as in variants one and three.

**Table 7:**
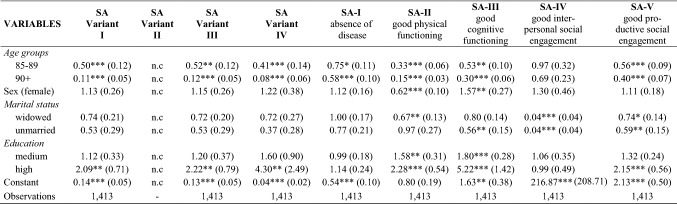
Results of multivariate logistic regressions for objective measurement of global SA and single dimensions

Odds Ratios, person calibration weights and clustering, standard errors in parentheses

***p < 0.001, **p < 0.05, *p < 0.01, *Variant I*: binary coding for all five dimensions; *Variant II*: upper third measurement concerning dimension social engagement not computable (n.c.), because the dependent variable has too little variance; *Variant III*: without criterion “paid work” concerning dimension productive social engagement (binary measurement); *Variant IV*: without criterion “paid work” concerning dimension productive social engagement, but with upper third measurement

When analysing the correlations with the individual dimensions of SA, there are interesting differences to the global measurement: Increasing age significantly reduces the absence of diseases. Male sex predicts a significantly higher probability of good physical functionality while female sex and being widowed correlate significantly with a good cognitive functionality. However, being widowed significantly reduces the probability of high physical functionality and high interpersonal social engagement. This also applies to unmarried or divorced individuals. Higher levels of education are significantly associated with high physical and cognitive functionality as well as with high productive social engagement.

The results of the multivariate linear regression analyses for subjective measurement of SA are presented in Table 8. Life satisfaction decreases with increasing age and among unmarried individuals but not in those with higher education. Positive experience of ageing is also significantly declining with increasing age while rising with a higher level of education. Affective well-being significantly decreases in older age, but is higher among women and individuals with higher levels of education. Lastly, valuation of life is significantly worse in higher age, for females and unmarried individuals, but better among persons with higher educational attainment. The correlations are in line with the results of the logistic regressions. No unexpected differences to the objective markers of SA are noticeable.

**Table 8:**
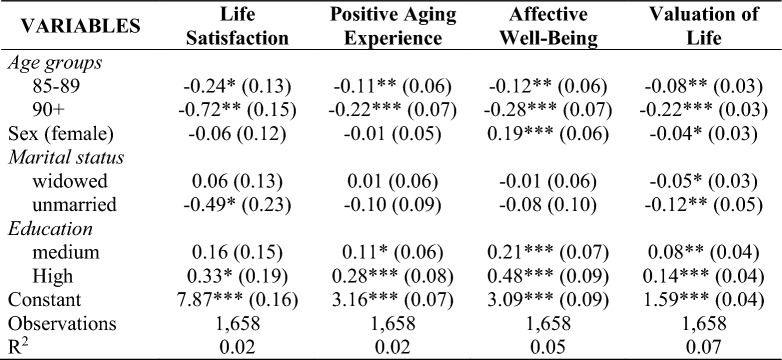
Results of Multivariate Linear Regression for Subjective Criteria of SA

Coefficients, person calibration weights and clustering, standard errors in parentheses

***p < 0.001, **p < 0.05, *p < 0.01

## Conclusion

The main focus of this study was to investigate SA empirically with a holistic view on objective and subjective markers among the oldest old, a mostly unexplored age segment so far. Distributions of different variants of operationalizations were calculated in order to critically evaluate definitions of SA. It became clear that it is necessary applying different markers of success to very old individuals in comparison to younger age groups, e.g., concerning productive social outcomes.

According to empirical tests of the standard definition of Rowe and Kahn (1997), 9% age successfully and only 2% pathologically. The distribution of the successful agers in the oldest old is comparable with the prevalence of successfully ageing people (85 +) in the Netherlands (von Faber et al. 2001). The finding is also in line with an assumption of a lower rate (< 12%) compared to the third age and meets the a priori criterion of 10% successful agers of a normal distribution. Furthermore, four to five criteria are fulfilled by a third. With regard to the dimensions of SA, it can be stated that most respondents report interpersonal and productive participation in social life despite a high burden of disease and cognitive as well as physical impairments. Nevertheless, multimorbidity and functional impairment pose a risk for the preservation of social contacts and societal contribution.

The logistic regression analyses show that younger age and higher educational attainment significantly increase the probability of SA, whereas sex and marital status are no significant correlates. This is in line with the results of the linear regression analyses (see Table 8) and mostly in line with the results of SA correlates by Thoma et al. (2020). However, the high values of subjective ratings reveal a remarkable discrepancy compared to objective criteria (see “Bivariate analyses of objective and subjective criteria for SA” section). The cross-tabulation of measurement modes confirms this assumption, too (see Table 6).

## Discussion

Staying healthy, living autonomously and leading a productive life may be unrealistic goals in the oldest old (Ribeiro and Araújo 2019). These dimensions proposed by Rowe and Kahn (1997) should be complemented by markers that holistically represent the value perception.

In view of that, a global SA indicator that is oriented by the development of the new Active Ageing-Well Being Index (Fritzell et al. 2020) might be useful. This index examined trends and inequality in a case study of the Swedish Panel Study of Living Conditions (75 +). Using analogies of this development can contribute to a discussion on a global indicator to explain inequalities of ageing processes. This indicator needs to weight objective and subjective criteria according to theoretical and empirical foundations. It should be considered whether results are based on subjectively assessed SA processes or whether it is more appropriate to measure the processes objectively through reduced social costs or decreased number of relatives in need of care.

Nonetheless, success is based on personality factors and the resilience to better adapt to life’s challenges as proposed by Pocnet et al. (2020). They underscore new prevention approaches with focus on inter- and intraindividual differences. Additionally, Calasanti and King (2020) advocate for a paradigm towards highlighting the role of personal choice and the need for normalizing old ages, likewise to react on accumulated inequality. It could be worth promoting strategies of adaptability to increase the possibilities to thrive in old age. Further research would contribute to a basis for intervention studies to support ageing processes as early as possible in the life-course. For instance, if one goal is to understand the needs required to support our current population of the oldest old, it should be concerned how many are able to be independent.

Finally, terms of “successfulness” of ageing in political or societal contexts should only be used critically. Nevertheless, the advantage of *successful* ageing prevails as a discourse catalyst of discussions about improvements in living conditions and quality of life of the oldest old.

### Strengths and limitations

Considering the high average age of this study, the response rate of 23% is very good compared to 27% reached in the German Ageing Survey (Klaus et al. 2017). Due to lack of data for the oldest old up to now, these analyses constitute a major contribution to gaining insights into their quality of life.

However, this study has certain limitations. Although the focus was to establish an operationalization for SA that responds to previous criticism, the final choice of measurements may influence estimates and relationships. Additional, in particular longitudinal data, are needed to provide a more solid basis to examine ageing as a process.

With regard to operationalization decisions, the dimension of productive social commitment was the greatest challenge, e.g., regarding association membership. It only indicates whether individuals are members of an association, but not their actual activity as members. Voluntary work, like paid work, is rather rare among the respondents (13%). The benchmark for this criterion remains unclear.

The discrepancy of objective and subjective indicators of SA can be explained by adaptation processes of ageing. Ribeiro and Araújo (2019) have defined success in the longevity by a scope review. They concluded a need for more constructs that include psychological aspects of adaptation. Unfortunately, indicators measuring adaptation mechanism that are acquired over the life course have not been examined. Future studies should test the successful life conduct by Wagner et al. (2018b) as alternative method.

## Supplementary Information

Below is the link to the electronic supplementary material.

Supplementary file1 (DOC 21 KB)

## Data Availability

The data has been transferred to Gesis and will be made available as soon as possible. The DOI will be submitted subsequently in the further publication process.
