# Supplementary material for: Successful ageing in the oldest old: objectively and subjectively measured evidence from a population-based survey in Germany
Source: Eur J Ageing. 2021 Mar 29;18(4):537–47. doi: 10.1007/s10433-021-00609-7 (PMC8563904; doi:10.1007/s10433-021-00609-7)
Supplement: Supplementary file 1 — Supplementary file1 (DOC 21 KB) [file 10433_2021_609_MOESM1_ESM.docx]

**Supplementary file**

**Table S1:** Operationalization and Distribution of Single Index Variables of Variant I

| SA-Domains | Final operationalization NRW80+ (Wagner et al., 2018b) |
| --- | --- |
| absence of disease | **Not having**   - coronary heart disease (93 %) - cardiac insufficiency (65 %) - stroke (92 %) - chronic obstructive pulmonary disease (COPD) (87 %) - cancer (excluding skin) (92 %) - diabetes (84 %) - Parkinson's (90 %) - absence of depression (73 %) |
| high physical function | **Independence in 14 ADLs/IADLs** (2 = no help, 1 = a little help, 0 = only with help)   - help with eating (M: 1.9, SD: 0.01) - help with dressing and undressing (M: 1.7, SD: 0.02) - help with body care (M: 1.6, SD: 0.02) - help with walking (M: 1.3, SD: 0.02) - help with getting up from bed and lying down (M: 1.8, SD: 0.02) - bathing or showering (M: 1.4, SD: 0.02) - reaching the toilet in time (M: 1.8, SD: 0.01) - using the phone (M: 1.8, SD: 0.01) - organize routes outside the running range (M: 1.4, SD: 0.02) - buy food and clothing yourself (M: 1.3, SD: 0.02) - preparing your own meals (M: 1.4, SD: 0.02) - do housework (M: 1.1, SD: 0.02) - medication (M: 1.5, SD: 0.02) - settlement of financial matters (M: 1.3, SD: 0.02) |
| high cognitive function | **Results from Demtect** (Kessler et al., 2012)   - normal (74 %) - mild cognitive impairment (16 %) - kind of dementia (1 %)   **Estimates of persons unable to provide information**   - no cognitive decline (10 %) - very low cognitive memory loss (9 %) - low cognitive losses (7 %) - moderate cognitive decline (12 %) - moderate severe cognitive decline (23 %) - severe cognitive decline (30 %) - very severe cognitive decline (9 %) |
| high interpersonal  social engagement | **Social activity**   - Sports (47 %) - coffee party (47 %) - concert, theatre, museum (29 %) - artistic activity (20 %) - further training (9 %)   **Social** **contact** (frequency)   - never (0.9 %) - seldom (14 %) - sometimes (26 %) - frequently (43 %) - very common (16 %)   **Living with others** (56 %) |
| high productive  social engagement | - paid work (3 %) - volunteering (13 %) - supporting others (49 %)   - helped with tasks or accomplishments of others (never: 51 %, seldom: 13 %, sometimes: 18 %, frequently: 14 %, very common: 4 %)   - comforted (never: 25 %, seldom: 19 %, sometimes: 31 %, frequently: 21 %, very common: 3 %) - association membership (25 %) |

*Note:* weighted data
